# Betaine Supplementation in Maternal Diet Modulates the Epigenetic Regulation of Hepatic Gluconeogenic Genes in Neonatal Piglets

**DOI:** 10.1371/journal.pone.0105504

**Published:** 2014-08-25

**Authors:** Demin Cai, Yimin Jia, Haogang Song, Shiyan Sui, Jingyu Lu, Zheng Jiang, Ruqian Zhao

**Affiliations:** Key Laboratory of Animal Physiology & Biochemistry, Nanjing Agricultural University, Nanjing, Jiangsu, P. R. China; University of Arkansas for Medical Sciences, United States of America

## Abstract

In this study, gestational sows were fed control or betaine-supplemented diets (3 g/kg) throughout the pregnancy, and the newborn piglets were used to elucidate whether maternal dietary betaine affected offspring hepatic gluconeogenic genes through epigenetic mechanisms. Neonatal piglets born to betaine-supplemented sows had significantly higher serum and hepatic betaine contents, together with significantly greater expression of methionine metabolic enzymes in the liver. Interestingly, significantly higher serum concentrations of lactic acid and glucogenic amino acids, including serine, glutamate, methionine and histidine, were detected in the piglets born to betaine-supplemented sows, which were coincident with higher hepatic glycogen content and PEPCK1 enzyme activity, as well as greater protein expression of gluconeogenic enzymes, pyruvate carboxylase (PC), cytoplasmic phosphoenolpyruvate carboxykinase (PEPCK1), mitochondrional phosphoenolpyruvate carboxykinase (PEPCK2) and fructose-1, 6-bisphosphatase (FBP1). Moreover, maternal betaine significantly changed the methylation status of both CpGs and histones on the promoter of gluconeogenic genes. The lower *PEPCK1* mRNA was associated with DNA hypermethylation and more enriched repression histone mark H3K27me3, while the up-regulated *PEPCK2* and *FBP1* mRNA was associated with DNA hypomethylation and more enriched activation histone mark H3K4me3. Furthermore, the expression of two miRNAs predicted to target *PC* and 6 miRNAs predicted to target *PEPCK1* was dramatically suppressed in the liver of piglets born to betaine-supplemented sows. Our results provide the first evidence that maternal betaine supplementation affects hepatic gluconeogenic genes expression in newborn piglets through enhanced hepatic methionine metabolism and epigenetic regulations, which involve DNA and histone methylations, and possibly miRNAs-mediated post-transcriptional mechanism.

## Introduction

Gluconeogenesis is an important metabolic pathway for endogenous glucose generation from substrates such as lactic acid and glucogenic amino acids [1]. Under certain circumstances, such as prolonged starvation, exercise or stress, gluconeogenesis is critical for the disposal of lactate and the maintenance of glucose homeostasis [2]. For newborns before suckling, gluconeogenesis has special physiological significance as it is the major source of glucose needed to confront parturition stress and to maintain tissue functions [3].

The rate of gluconeogenesis is controlled by key enzymes including pyruvate carboxylase (PC), phosphoenolpyruvate carboxykinase (PEPCK1) (cytosolic PEPCK1 and mitochondrial PEPCK2), fructose-1, 6-bisphosphatase (FBP1), and glucose 6-phosphatase (G6PC) [4]. A slew of studies have demonstrated that these gluconeogenic enzymes are highly vulnerable to maternal nutrition [5], [6]. Furthermore, the nutritional programming of offspring gluconeogenesis involves epigenetic regulations such as DNA methylation, histone modifications and microRNA-mediated post-transcriptional regulation [7]. Methyl donors, such as methionine or folic acid, are able to reverse the epigenetic modifications and thereby restore the behavioral or metabolic disorders in offspring caused by prenatal or neonatal adverse experiences [8], [9].

Betaine functions as a methyl donor to convert homocysteine to methionine in a reaction catalyzed by betaine homocysteine methyltransferase (BHMT) [10]. Methionine is then converted to S-adenosylmethionine (SAM) by methionine adenosyl transferase (MAT) [11]. SAM acts as a methyl donor for DNA and protein methylation which is critical for the epigenetic regulation of gene expression. After donating its methyl group to acceptor molecules, SAM is converted to S-adenosylhomocysteine (SAH) which is then hydrolyzed to homocysteine by S-adenosylhomocysteine hydrolase (AHCY). It has been shown that SAM: SAH ratio affects DNA methylation in general and elevated SAM: SAH ratio correlates with global DNA hypermethylation [12].

Betaine is derived from either choline oxidation or dietary intake, and is critical for embryonic and fetal development [13]. Betaine deficiency is associated with a number of metabolic disorders [13]. Dietary supplementation of betaine can prevent obesogenic diet-induced hepatic steatosis and reduce obesogenic diet-induced fatty liver [14]–[16]. Betaine supplementation also improves the growth performance and carcass characteristics in domestic animals [17], [18], yet the mechanisms remain unclear. It is suggested that the hepatoprotection of betaine may be achieved through its effect on hepatic gluconeogenesis [19]. However, direct evidence regarding the effects of betaine on hepatic gluconeogenesis is lacking. Moreover, the effect of betaine supplementation in maternal diet during gestation on hepatic gluconeogenesis in neonatal offspring has not been investigated.

Therefore, here we use pigs as model to investigate whether feeding gestational sows with betaine-supplemented diet may affect hepatic gluconeogenesis in newborn piglets. To explore the possible epigenetic mechanisms underlying such effects, we detected hepatic expression of genes involved in gluconeogenesis and methionine metabolism, as well as the status of DNA methylation and histone modifications on the promoter of gluconeogenic genes, together with the expression of microRNAs which are predicted to target gluconeogenic genes in the liver of neonatal piglets.

## Materials and Methods

### Animals and samples

Landrace×Yorkshire crossbred sows in the second parity were artificially inseminated, at the observation of estrus, with a mixture of Duroc semen samples obtained from two littermate boars. One week after the artificial insemination, sows were randomly divided into control and betaine groups (8 per group). Sows in control group received basal diet while those in betaine group were fed betaine-supplemented (3 g/kg) diet throughout the pregnancy. Betaine was in the form of betaine hydrochlorides with 98% purity, purchased from SKYSTONE FEED CO., LTD (Jiangsu, China). The diet composition is shown in Table S1. All sows were housed at 25°C with 50% of humidity on a 12 h/12 h light/dark cycle. Sows were fed three times a day at 05:00, 10:00 and 17:00 h, and had free access to water. Newborn piglets were individually weighed immediately after parturition. The piglets of the same litter were kept together in the warm creep area. There were altogether 16 litters of piglets, 8 litters in each group. One male and one female piglet of the mean body weight (± 10%) were selected from each litter and exsanguinated before suckling. Blood was collected immediately and the liver (without the gall bladder) was harvested within 20 min, snap-frozen in liquid nitrogen, and stored at -80°C for further analysis.

The experimental protocol was approved by the Animal Ethics Committee of Nanjing Agricultural University, with the project number 2012CB124703. The slaughter and sampling procedures complied with the “Guidelines on Ethical Treatment of Experimental Animals” (2006) No. 398 set by the Ministry of Science and Technology, China.

### Serum concentration and hepatic content of betaine

Frozen serum and liver samples of the newborn piglets were shipped to the China National Feed Quality Control Center, Chinese Academy of Agricultural Sciences, Beijing, China and were prepared for the determination of betaine as described previously [20]. Betaine concentrations in serum and liver samples were measured with an liquid chromatography (Aglient 1200, Aglient Techologies)–mass spectrometry (API 5000TM, AB SCIEX) system optimized for the measurement of betaine in animal samples.

### Serum biochemical metabolites, hormones and amino acids profile

Serum concentrations of biochemical metabolites, including glucose and lactic acid, were detected with the enzymatic colorimetric methods using commercial kits for glucose (no. 6006, Shanghai Rongsheng Biotech) and lactic acid (no. A019, Nanjing Jiancheng Bioengineering Institute). Serum concentrations of insulin and glucagon were measured with respective commercial RIA kits (nos. F01PZB and F03PZB, Beijing North Institute of Biological Technology) with assay sensitivities of 0.29 pmol/L and 16.1 ng/L, respectively. The intra- and inter-assay variations were 10 and 15%, respectively, for both assays.

Serum samples for measuring the free amino acids concentrations were prepared according to a previous publication [21]. Serum concentrations of free amino acids were determined with an automatic amino acid analyzer (L-8900, Hitachi, Japan) in duplicate. The intra- and inter-assay coefficients of variation were 3 and 6%, respectively.

### Liver glycogen content

The hepatic glycogen content was determined as previously described [22]. The results are expressed as mg glycogen/g liver (wet weight).

### Real-time RT-PCR for mRNA quantification

Total RNA was isolated from liver samples using TRIzol Reagent (no. 15596026, Invitrogen) according to the manufacturer's instruction and reverse transcribed with the PrimeScript 1st Strand cDNA Synthesis kit (no. D6110A, Takara). Two microliters of diluted cDNA (1∶25) were used in each real-time PCR assay with Mx3000P (Stratagene). It is a routine practice to confirm the absence of genomic DNA contamination in samples used for Real Time PCR analysis. Firstly, RNA samples were treated with DNase before reverse transcription to cDNA; secondly, DNase-treated RNA samples were directly used as template for QPCR to ensure that there was no specific amplification; thirdly, primers were designed to span an intron so any genomic DNA contamination can be easily reported by an extra PCR product in melting curve analysis. Peptidylprolyl isomerase A (PPIA) was chosen as a reference gene, because it is expressed in similar abundance to the genes of interest and its expression was not affected by the experimental factor. All primers were synthesized by Generay Biotech and listed in Table S4.

### Western Blotting for protein quantification

Liver samples were homogenized in RIPA buffer (50 mM Tris-HCl pH 7.4, 150 mM NaCl, 1% NP40, 0.25% Na-deoxycholate, 1 mM PMSF, 1 mM sodium orthovanadate with Roche EDTA-free complete mini protease inhibitor cocktail, no. 11836170001). Protein concentrations were determined with a Pierce BCA Protein Assay kit (no. 23225, Thermo). Western-blot analysis for target proteins was carried out according to the protocols provided by the primary antibody suppliers. The sources of the primary antibodies used in Western blotting are listed in Table S5. GAPDH or β-actin was selected as loading control.

### Enzyme assay for PEPCK1 activity

Hepatic PEPCK1 enzyme activity was detected as previously described with minor modifications [23]. In brief, 0.5 g liver tissue was homogenized in the lysis buffer (0.25 mol/L sucrose and 5 mmol/L Tris-HCl, pH 7.4) at 4°C. The liver lysates were centrifuged at 3500 g for 15 min, and the supernatant was collected for a further centrifugation (11000 g) for 40 min. Cytosolic supernatant containing 0.2 mg of total protein was added to a 1 mL reaction buffer (50 mmol/L Tris-HCl, 50 mmol/L NaHCO3, 1 mmol/L MnCl2, 1 mmol/L phosphoenolpyruvate, 2 U malate dehydrogenase, and 0.25 mmol/L tinamide adenine dinucleotide. Finally, 2′-deoxyguanosine 5′-diphosphate at a 0.15 mmol/L concentration was added to start the reaction, and the decrease in absorbance at 340 nm in 4 min was measured.

### Methylated DNA immunoprecipitation (MeDIP) analysis

High-quality genomic DNA isolated from liver was sonicated to produce small fragments ranging from 300 to 1000 bp. Two micrograms of fragmented DNA were heat denatured to produce single-stranded DNA. A mouse monoclonal antibody against 5-methyl cytidine (ab10805, Abcam) was used to immunoprecipitate the methylated DNA fragments. The immune complexes were captured with protein G agarose beads (40 µL, 50% slurry, pretreated with denatured salmon sperm DNA and BSA, sc-2003, Santa Cruz Biotechnology). The beads bound to immune complexes were washed to eliminate nonspecific binding and resuspended in 250 µL digestion buffer containing proteinase K. Finally, the MeDIP DNA was purified. A small aliquot of MeDIP DNA was used to amplify the proximal promoter sequences of the target genes by real-time PCR. A pair of negative control primers is used to amplify a fragment of *FBP1* promoter absent of CpG sites. MeDIP results were calculated relative to the negative control and presented as the fold change relative to the average value of control group. The specific and negative control primers were designed with Primer 5 software and shown in Table S4.

### Chromatin immunoprecipitation (ChIP) assay

ChIP analysis was performed according to our previous publication [24] with some modifications. Firstly, frozen liver samples (approximate 200 mg) were ground in liquid nitrogen and resuspended with PBS containing protease inhibitor cocktail (no. 11697498001, Roche). Formaldehyde was added to a final concentration of 1% for cross-linking protein and DNA, then glycine was added (2.5 mol/L) to stop the reaction at room temperature. Following centrifugation, the pellets were rinsed with PBS and lysed in SDS lysis buffer containing protease inhibitors. The crude chromatin preparations were sonicated to an average length ranging from 200 to 500 bp and precleared with salmon sperm DNA-treated protein G agarose beads (40 µL, 50% slurry, sc-2003, Santa Cruz Biotechnology). The precleared chromatin preparations were incubated with 2 µg of respective primary antibody overnight at 4°C (Antibodies information is shown in Table S5). A negative control was included with normal rat IgG. Protein G agarose beads (40 µL, 50% slurry, sc-2003, Santa Cruz Biotechnology) were added to capture the immunoprecipitated chromatin complexes. Finally, DNA fragments were released from the immunoprecipitated complexes via reverse cross-linking at 65°C for 1 h and were purified. Immunoprecipitated DNA was quantified by real-time PCR. Also, CpG islands were predicted in the porcine gluconeogenic gene promoters by Sequence Manipulation Suite [25]. Possible transcriptional factors binding sites were predicted by TRANSFAC 6.0. (GR) and glucocorticoid receptor-element (GRE) sites were predicted on each promoter region of relative genes. All the specific primers were shown in Table S4.

### Quantification of miRNAs targeting PC and PEPCK1

Two micrograms of total RNA treated with RNase-free DNase I (Promega) was polyadenylated by poly (A) polymerase using Poly (A) Tailing Kit (AM1350, Applied Biosystems, USA) according to the manufacturer's instruction. Polyadenylated RNA was then dissolved and reverse transcribed using poly (T) adapter. Real-time PCR was performed with SYBR green qPCR master mix reagent (TaKaRa, Japan) in triplicates with a miRNA specific forward primer and a universal reverse primer complementary to part of the poly (T) adapter sequence. *U6* small nuclear RNA (*U6* snRNA) was used as a reference gene to normalize the expression of miRNAs.

Because the 3'UTR sequence of porcine *PC* gene has not been reported, we aligned the 3′ flanking sequence of this gene with the 3'UTR sequence of human *PC* gene to obtain the consensus sequence for miRNA prediction using an online miRNAs prediction tool, PITA algorithm, with the threshold of score set at -10 [26]. Twenty miRNAs were predicted to target PC and 15 miRNAs predicted to target PCK1. Among all these predicted miRNAs, 7 miRNAs for each gene were reliably and repeatedly quantified in real-time PCR. The primer sequences used for miRNAs analysis are listed in Table S6

### Statistical analysis

Data are presented as means ± SEM. When males and females were analyzed separately, control for litter was not necessary because they were all from different litters. Since none of the detected parameters showed sex disparity, we pooled male and female together making n = 16 for each group. Comparisons were made using two-tailed Student's t test for independent data. The 2^−△△Ct^ method was used to analyze real-time PCR data. The relative quantifications of mRNA, protein, CpG methylation, histone modifications, and miRNA were expressed as the fold change relative to the mean value of control group. All experiments were carried out in triplicate. The differences were considered statistically significant when *P*<0.05.

## Results

### Reproductive performance and serum glucogenic amino acids concentration of sows

Maternal betaine supplementation had no effects on litter size or litter weight (Table S2). Serum concentrations of glucogenic amino acids, including serine, glutamate, methionine and histidine, in sows were not affected by dietary betaine supplementation. Serum arginine concentration was below the detection limit (Table S3).

### Serum concentrations of betaine, hormones and metabolites in piglets

Piglets born to betaine-supplemented sows exhibited significantly higher serum betaine concentration (*P*<0.05) as compared to their control counterparts. Body weight and liver weight did not differ, nor did the serum concentrations of glucose, insulin, glucagon or the ratio of insulin to glucagon. However, serum concentrations of lactate (*P*<0.05) and glucogenic amino acids (*P*<0.05), including serine, glutamate, methionine and histidine, were all significantly higher in piglets born to betaine-supplemented sows than that from the control group. Moreover, piglets born to betaine-supplemented sows tended to have higher (*P* = 0.08) serum arginine concentration (Table 1).

**Table 1:** Body and liver weight, betaine concentration in serum and liver, hepatic glycogen content, biochemical metabolites, hormones and amino acids in serum of newborn piglets.

Variables	Control (n = 16)	Betaine (n = 16)
Body weight, kg	1.56±0.05	1.61±0.07
Liver weight, g	40.9±1.93	44.9±2.90
Glycogen, g/g	0.13±0.01	0.16±0.01*
Serum betaine, μmol/L	4.01±0.41	5.39±0.37*
Hepatic betaine, μmol/g	0.61±0.09	0.90±0.07*
Biochemical metabolites		
Glucose, mmol/L	3.20±0.35	2.64±0.73
Lactic acid, mmol/L	5.12±0.27	6.29±0.38*
Hormones		
Insulin, pmol/L	4.51±0.91	5.33±0.82
Glucagon, μg/L	0.32±0.04	0.26±0.02
Insulin/Glucagon	0.12±0.03	0.14±0.02
Amino acids		
Arginine, μmol/L	11.8±4.73	26.9±6.28
Glutamate, mmol/L	0.21±0.02	0.33±0.04*
Histidine, μmol/L	36.3±7.17	57.6±6.82*
Methionine, μmol/L	6.86±1.05	15.1±3.20*
Serine, mmol/L	0.14±0.01	0.18±0.01*

Values are means ± SEM, n = 16 (8 males plus 8 females).

*Different from Control, P<0.05.

### Hepatic betaine content and expression of methionine metabolic genes

**Figure 1:**
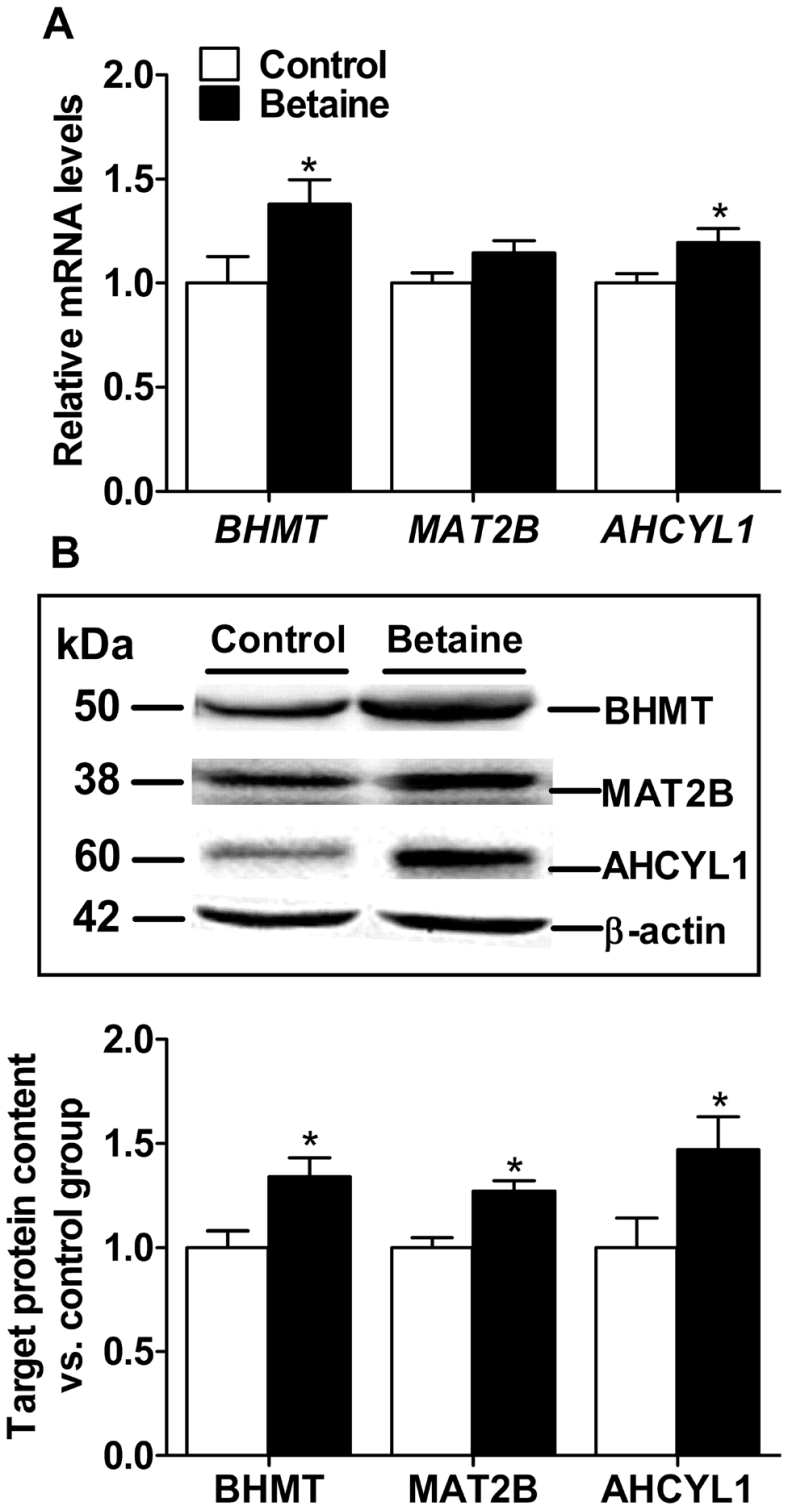
Hepatic mRNA abundance of genes (A), protein content and western blot bands for relevant proteins (B) involved in methionine metabolism in newborn piglets. Values are means ± SEM, n = 16 (8 males plus 8 females). Different from control, ^*^ *P*<0.05. AHCYL1, adenosylhomocysteine hydrolase-like 1; BHMT, betaine-homocysteine methyltransferase; MAT2B, methionine adenosyltransferase II beta.

Piglets born to betaine-supplemented sows had significantly higher betaine content (*P*<0.05) in the liver (Table 1), which was associated with significant up-regulation of methionine metabolic genes. *BHMT* and *AHCYL1* were significantly up-regulated (*P*<0.05), while *MAT2B* tended to be higher (*P* = 0.06), at the level of mRNA (Figure 1A). In accordance with the mRNA abundance, the protein content of BHMT, AHCYL1 and MAT2B was all remarkably greater (*P*<0.05) in the liver of piglets born to betaine-supplemented sows (Figure 1B).

### Hepatic glycogen content and PEPCK1 enzyme activity, and expression of gluconeogenic genes

**Figure 2:**
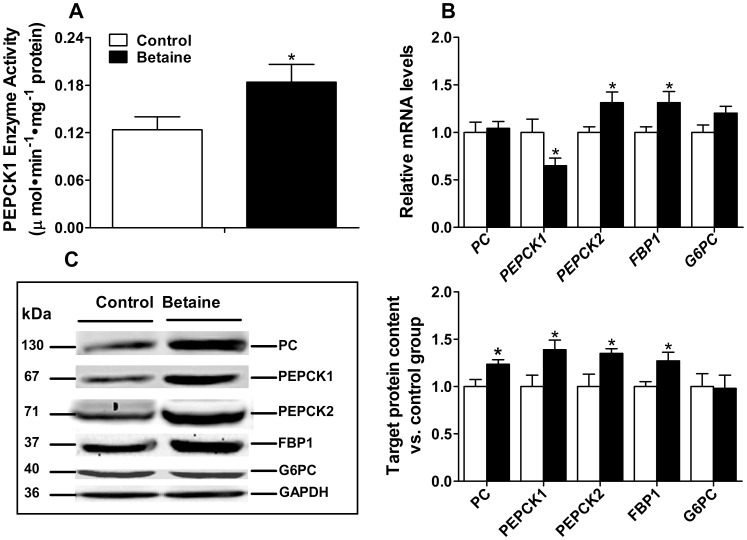
Hepatic PEPCK1 enzyme activity (A), mRNA abundance of genes (B), protein content and western blot bands for relevant proteins (C) involved in gluconeogenesis and in newborn piglets. Values are means ± SEM, n = 16 (8 males plus 8 females). Different from control, ^*^ *P*<0.05. FBP1, fructose-1, 6-bisphosphatase; G6PC, glucose-6-phosphatase; PC, pyruvate carboxylase; PEPCK1, phosphoenolpyruvate carboxykinase 1; PEPCK2, phosphoenolpyruvate carboxykinase 2.

Hepatic glycogen content was significantly higher (*P*<0.01) (Table 1), and hepatic PEPCK1 enzyme activity was 0.5 fold higher in the piglets born to betaine-supplemented sows compare to that of control piglets (Figure 2A). Accordingly, hepatic expression of gluconeogenic genes, *PEPCK2* and *FBP1*, were significantly up-regulated (*P*<0.05) in the piglets born to betaine-supplemented sows, at both mRNA and protein levels (Figure 2B and C). PC and PEPCK1 were also up-regulated at protein expression (*P*<0.05) (Figure 2C), yet with uncoupled mRNA expression (Figure 2B). *G6PC* tended to be higher (*P* = 0.07) at the level of mRNA, but not protein, whereas PC was significantly higher (*P*<0.05) at the level of protein, but not mRNA, in the liver of piglets born to betaine-supplemented sows. *PEPCK1* demonstrated reversed alterations for mRNA that was significantly lower (*P*<0.05), and protein that was significantly higher (*P*<0.05), in the liver of piglets born to betaine-supplemented sows (Figure 2B and C). The uncoupled mRNA and protein expression implicates possible involvement of post-transcriptional mechanism in gluconeogenic gene regulation.

### Epigenetic modifications and GR binding of gluconeogenic gene promoters

**Figure 3:**
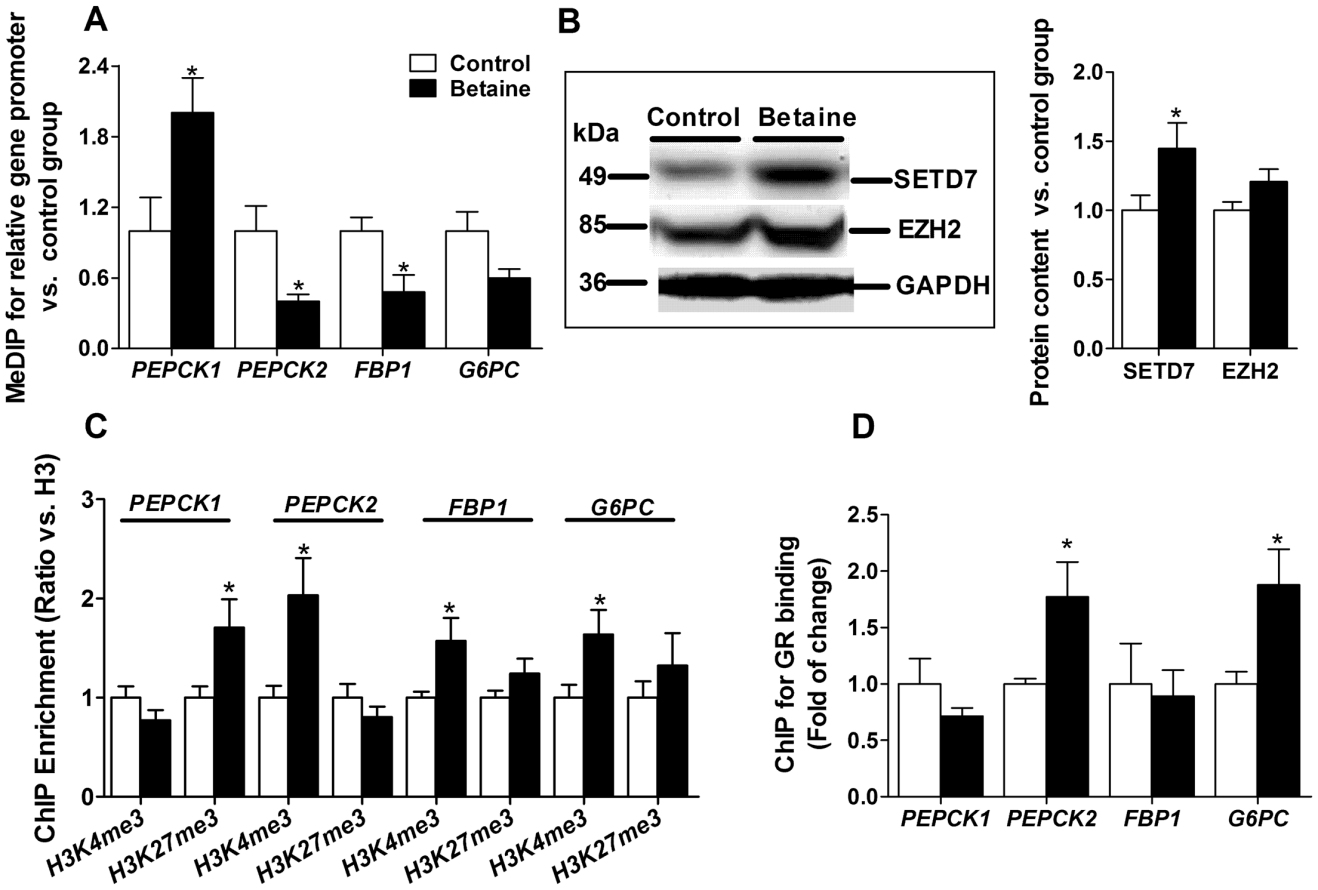
DNA methylation at *PEPCK1*, *PEPCK2*, and *FBP1* and *G6PC* promoter in liver of newborn piglets (A). B: Western blotting results and relevant western blot bands of histone methyltransferases SETD7 and EZH2. C: ChIP analysis of histone modifications in the promoter of hepatic *PEPCK1*, *PEPCK2*, and *FBP1* and *G6PC* normalized to total histone H3 respectively in newborn piglets. D: ChIP analysis of GR binding in the promoter of hepatic *PEPCK1*, *PEPCK2*, and *FBP1* and *G6PC*. Values are means ± SEM, n = 16 (8 males plus 8 females). Different from control, ^*^ *P*<0.05. EZH2, enhancer of zeste homolog 2; FBP1, fructose-1, 6-bisphosphatase; G6PC, glucose-6-phosphatase; GR, glucocorticoid receptor; PEPCK1, phosphoenolpyruvate carboxykinase 1; PEPCK2, phosphoenolpyruvate carboxykinase 2; SETD7, SET domain-containing protein 7.

MeDIP analysis revealed significant a hypomethylation (*P*<0.05) on the promoter of *PEPCK2* and *FBP1* genes, which was reversely correlated to the up-regulation of these two genes in mRNA expression. Interestingly, the level of CpG methylation on *G6PC* promoter tended to be lower (*P* = 0.07) in the liver of piglets born to betaine-supplemented sows corresponding to the trend of higher *G6PC* mRNA expression. In contrast, *PEPCK1* promoter was significantly hypermethylated (*P*<0.05), which was in accordance with the diminished *PEPCK1* mRNA expression in the liver of piglets born to betaine-supplemented sows (Figure 3A).

The enrichment of two histone modification marks, the activation mark histone H3 lysine 4 trimethylation H3K4me3 and the repression mark histone H3 lysine 27 trimethylation (H3K27me3) on the promoter of gluconeogenic genes was determined with ChIP assay using specific antibodies. The enrichment of histone marks is normalized with that of histone H3. As shown in Figure 3C, hepatic inhibition of *PEPCK1* gene transcription in the piglets born to betaine-supplemented sows was associated with an increment of the repression mark H3K27me3 (*P*<0.05), while hepatic activation of *PEPCK2*, *FBP1* and *G6PC* (Figure 3C) was accompanied with significantly more enriched activation mark H3K4me3 (*P*<0.05) on the promoters.

The lysine methyltransferase SETD7 which trimethylates histone H3 lysine 4 (H3K4) was significantly up-regulated (*P*<0.05, Figure 3B), while the lysine methyltransferase EZH2 which trimethylates H3 lysine 27 (H3K27) [27] tended to be higher (*P* = 0. 07, Figure 3B), in the liver of piglets born to betaine-supplemented sows at the protein level.

Besides, the ChIP assay revealed higher (P<0.05) GR binding to *PEPCK2* and *G6PC* gene promoter in betaine-exposed piglet liver, but no difference was detected in *PEPCK1* and *FBP1* (Figure. 3D).

### Expression of microRNAs predicted to target PC and PEPCK1

**Figure 4:**
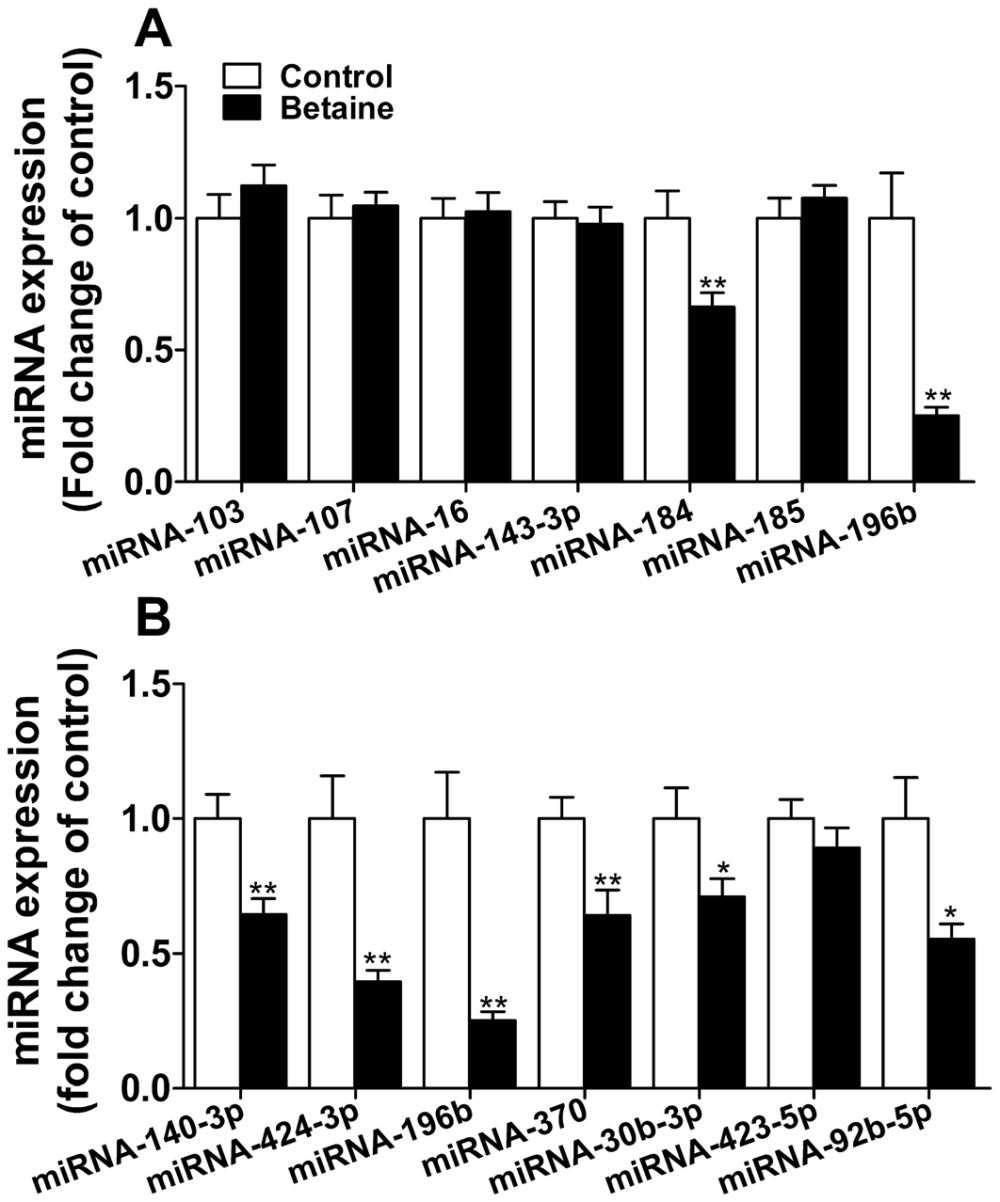
MicroRNAs targeting *PC* (A) and *PEPCK1* (B) 3′UTR in the liver of newborn piglets. Values are means ± SEM, n = 16 (8 males plus 8 females). Different from control, ^*^ *P*<0.05 and ^**^ *P*<0.01. PC, pyruvate carboxylase; PEPCK1, phosphoenolpyruvate carboxykinase 1.

To explore whether post-transcriptional mechanisms are involved in the regulation of *PC* and *PEPCK1*, we further detected hepatic expression of miRNAs predicted to target these 2 genes. Piglets born to betaine-supplemented sows demonstrated a significant down-regulation in the hepatic expression of miRNA-184 (*P*<0.01) and miRNA-196b (*P*<0.01), which are predicted to target *PC* (Figure 4A), and miRNA-1403p (*P*<0.01), miRNA-424-3p (*P*<0.01), miRNA-196b (*P*<0.01), miRNA-370 (*P*<0.01), miRNA-30b-3p (*P*<0.05) and miRNA-92b-5p (*P*<0.05), which are predicted to target *PEPCK1* (Figure 4B). Diminished expression of these regulatory miRNAs was in line with higher protein content of PC and PEPCK1 detected in the liver of piglets born to betaine-supplemented sows.

## Discussion

Betaine serves as a substrate for the formation of methionine which is not synthesized *de novo* in mammals [28], and it is noted that betaine significantly elevates serum methionine level in healthy adult men [29]. Moreover, previous study has shown that betaine can be actively transported across placenta from mother to fetus [30]. In the present study, serum concentration of methionine was elevated and higher betaine concentration was detected in the serum and the liver of betaine-exposed piglets. Betaine is also reported to raise serum serine concentration through folate-dependent remethylation reaction [31]. Therefore, higher methionine and serine detected in the betaine-exposed piglets could be the direct consequences of increased betaine concentration and metabolism, whereas the higher serum levels of glutamate, histidine and arginine may attribute to subsequent methionine metabolism and related pathways [32].

It is well known that lactate and glucogenic amino acids are substrates of hepatic gluconeogenesis. In this study, betaine-exposed piglets did not show significant alteration in serum glucose level, yet hepatic glycogen content was drastically higher compared to their control counterparts. This finding is in line with the report that betaine supplementation increases hepatic glycogen content by reducing glycogen synthetic rate-limiting enzyme GSK3α in mice [19], in spite of lower glucose production. Unfortunately, we were not able to detect the mRNA expression of *GSK3α* gene in the liver of neonatal piglets may result from that the porcine *GSK3α* mRNA sequence published online is predicted. Elevated serum concentrations of gluconeogenic substrates combined with higher hepatic glycogen content point to possible activation of hepatic gluconeogenic pathway. Indeed, we detected significant up-regulation of genes encoding key gluconeogenic pathway, at mRNA or/and protein levels, which is a reflection of methyl donors and their effects on gluconeogenic genes [33], [34].

Betaine donates methyl groups for protein and DNA methylation reactions through methionine metabolic pathway [28]. Previous investigations demonstrate that betaine supplementation causes BHMT up-regulation [35], [36]. However, in this study, all the three key enzymes involved in methionine metabolism, BHMT, MAT2B and AHCYL1, were up-regulated in the liver of newborn betaine-exposed piglets. Epigenetic modifications such as DNA methylation and histone modifications, using the methyl groups, play an important role in regulating gluconeogenic genes transcription. Feeding high energy diet to female rats at conception decreases hepatic *PEPCK* expression in offspring through modified DNA methylation in its promoter [6]. Moreover, maternal dietary protein restriction programs hepatic *G6PC* gene in newborn piglets which is associated with hypomethylation of *G6PC* gene promoter as well as changed H3K4me3 and H3K27me3 [7]. Interestingly, in the present study, *PEPCK1* gene promoter was found to be hypermethylated, whereas the promoters of *PEPCK2* and *FBP1* genes were hypomethylated in the liver of piglets prenatally exposed to betaine. Nevertheless, the levels of DNA methylation on promoters were reversely correlated with the mRNA abundances of respective genes.

Increased supply of methyl donors and enhanced methionine metabolism generally result in global DNA hypermethylation [37], yet do not necessarily cause hypermethylation on the promoter of all the functional genes. In this study, both hypermethylation (for *PEPCK1*) and hypomethylation (for *PEPCK2* and *FBP1*) were detected on the promoter of gluconeogenic genes in the liver of piglets born to betaine-supplemented sows. In line with our results, specific CpG sites in fatty acid synthase (*FASN*) gene promoter was found to be hypomethylated in the liver of betaine-supplemented rats [16]. Furthermore, gestational deficiency of choline, the major precursor of betaine, induces hypomethylation of the regulatory CpGs within the *DNMT1* gene, which results in the hypermethylation of global DNA [38]. These findings suggest that methyl donors modulate DNA methylation machinery in a complex gene-dependent manner.

SETD7 and EZH2 are SAM-dependent enzymes [27], [39], and suppression of SAM-dependent methylations causes reduction of SETD7 and EZH2, leading to lower level of H3K4me3 and H3K27me3 [40], [41]. Therefore, enhanced methionine metabolism may contribute to higher SETD7 and EZH2 protein contents, and the latter led to the higher enrichment of H3K27me3 in the *PEPCK1* promoter together with elevated H3K4me3 in the *PEPCK2*, *FBP1* and *G6PC* promoter in the present study. Moreover, it is noted that the detected CpG islands on gluconeogenic gene promoters are predicted to contain binding sites for GR and the results showed higher GR binding to hepatic *PEPCK2* and *G6PC* gene promoters in betaine-exposed piglet. In addition, previous studies demonstrate that gene promoters of *PEPCK* and *G6PC* contain positive GRE sites [42], [43], suggesting that chromatin remodeling caused by altered DNA and histone methylation status on *PEPCK2* and *G6PC* promoters may increase the binding of GR, thereby up-regulating the transcriptional level of the two genes.

Another interesting finding in the present study is the incongruity between the mRNA and protein levels for *PC* and *PEPCK1* genes. *PC* was unchanged and *PEPCK1* was even lower at mRNA levels, but both PC and PEPCK1 were greater at protein level. The dissociation of mRNA abundance and the protein content implies possible involvement of post-transcriptional regulation. MicroRNAs are known to participate predominantly in the post-transcriptional regulation through targeting mRNA degradation and/or translational repression. Previous studies indicate that miRNAs predominantly promote cleavage of mRNAs in plants [44], [45], while in animals miRNAs act mainly through translational repression [46]. Moreover, it has been reported that in animal models and cell lines, miRNAs target in principal the protein translation rather than mRNA degradation [47], [48]. Therefore, when transcriptional regulation and miRNA-mediated translational repression are not synchronized, mRNA and protein levels can be un-coupled. In this study, two out of 7 miRNAs targeting *PC* and 6 out of 7 miRNAs targeting *PEPCK1* were dramatically suppressed in the liver of piglets born to betaine-supplemented sows, suggesting inhibition of miRNAs-mediated translation repression for these two genes. Although this finding may hint the underlying mechanisms for the mismatched mRNA and protein expression of *PC* and *PEPCK1* genes, the roles of these miRNAs in regulating gluconeogenic genes in porcine liver await further functional verification.

In conclusion, betaine supplementation in maternal diet during gestation affects hepatic gluconeogenic genes in newborn piglets via epigenetic regulation including DNA methylation, histone modifications and miRNAs, which is associated with enhanced methionine metabolism. Neonatal changes in hepatic gluconeogenic gene expression may cause long-term consequences in glucose homeostasis later in adult life. Long-term follow-up studies are required to understand whether such fetal programming of hepatic gluconeogenic genes caused by maternal betaine supplementation is beneficial or detrimental for adult health.

## Supporting Information

Table S1 **Composition and nutrient content of the experimental diet.**
(DOC)

Table S2 **Reproductive performance of sows fed control or betaine supplemented diet as measured by littler size and littler weight.**
(DOC)

Table S3 **Amino acids concentration in serum of sows.**
(DOC)

Table S4 **Nucleotide sequences of specific primers.**
(DOC)

Table S5 **Antibodies for this experiment.**
(DOC)

Table S6 **miRNA and the corresponding primer sequences.**
(DOC)
