# Supplementary material for: Betaine Supplementation in Maternal Diet Modulates the Epigenetic Regulation of Hepatic Gluconeogenic Genes in Neonatal Piglets
Source: PLoS One. 2014 Aug 25;9(8):e105504. doi: 10.1371/journal.pone.0105504 (PMC4143294; doi:10.1371/journal.pone.0105504)
Supplement: Table S1 — Composition and nutrient content of the experimental diet. (DOC) [file pone.0105504.s001.doc]

**Table S1 Composition and nutrient content of the experimental diet**

|  | Control | Betaine |
| --- | --- | --- |
| Ingredient, *g/kg* |  |  |
| Corn | 370 | 370 |
| Wheat | 300 | 300 |
| Bran | 80 | 80 |
| Soybean meal | 170 | 170 |
| Lignocelluloses | 30 | 30 |
| CaHPO4 | 20 | 20 |
| Soybean oil | 8 | 8 |
| Premix* | 20 | 20 |
| Choline | 0.42 | 0.42 |
| Betaine | 0 | 3 |
| Digestible energy, *MJ/kg* | 13.1 | 13.1 |
| Calculated composition, % |  |  |
| Crude protein | 15 | 15 |
| Crude fiber | 4.5 | 4.5 |
| Calcium | 0.84 | 0.84 |
| Phosphorous | 0.65 | 0.65 |

* The premix contains (per kilogram): vitamin A: 240,000 IU; vitamin D-3: 60,000 IU; vitamin E: 720 IU; vitamin K-3: 30 mg; vitamin B-1: 30 mg; vitamin B-2: 120 mg; vitamin B-6: 60 mg; vitamin B-12: 360 mg; niacin: 600 mg; pantothenic acid: 300 mg; folic acid: 6 mg; manganese sulphate: 1.0 g; zinc oxide: 2.5 g; ferrous sulphate: 4.0 g; copper sulphate: 4.0 g; sodium selenite: 6 mg; calcium: 150 g; phosphorus: 15 g; sodium chloride: 40 g.
