# Supplementary material for: Betaine Supplementation in Maternal Diet Modulates the Epigenetic Regulation of Hepatic Gluconeogenic Genes in Neonatal Piglets
Source: PLoS One. 2014 Aug 25;9(8):e105504. doi: 10.1371/journal.pone.0105504 (PMC4143294; doi:10.1371/journal.pone.0105504)
Supplement: Table S2 — Reproductive performance of sows fed control or betaine supplemented diet as measured by littler size and littler weight. (DOC) [file pone.0105504.s002.doc]

**Table S2 Reproductive performance of sows fed control or betaine supplemented diet as measured by littler size and littler weight**

| Variables | Control (n = 8) | Betaine (n = 8) |
| --- | --- | --- |
| Litter size, n | 12.89 ± 0.72 | 12.22 ± 0.68 |
| Live litter size, n | 12.22 ± 0.55 | 11.67 ± 0.69 |
| Still birth rate | 0.05 ± 0.02 | 0.04 ± 0.02 |
| Litter weight, kg | 18.45 ± 0.75 | 19.35 ± 1.30 |

Values are mean ± SEM, n = 8/ group.
