# Supplementary material for: Betaine Supplementation in Maternal Diet Modulates the Epigenetic Regulation of Hepatic Gluconeogenic Genes in Neonatal Piglets
Source: PLoS One. 2014 Aug 25;9(8):e105504. doi: 10.1371/journal.pone.0105504 (PMC4143294; doi:10.1371/journal.pone.0105504)
Supplement: Table S3 — Amino acids concentration in serum of sows. (DOC) [file pone.0105504.s003.doc]

**Table S3 Amino acids concentration in serum of sows**

| Variables | Control (n = 8) | Betaine (n = 8) |
| --- | --- | --- |
| Arginine, μmol/L | ND | ND |
| Glutamate, mmol/L | 0.19 ± 0.02 | 0.21 ± 0.02 |
| Histidine, mmol/L | 0.23 ± 0.02 | 0.20 ± 0.04 |
| Methionine, μmol/L | 76.50 ± 3.89 | 57.11 ± 10.46 |
| Serine, mmol/L | 0.11 ± 0.10 | 0.11 ± 1.16 |

Values are mean ± SEM, n = 8/ group. ND, not detected. Serum arginine concentration was below the detection limit.
