# Supplementary material for: Betaine Supplementation in Maternal Diet Modulates the Epigenetic Regulation of Hepatic Gluconeogenic Genes in Neonatal Piglets
Source: PLoS One. 2014 Aug 25;9(8):e105504. doi: 10.1371/journal.pone.0105504 (PMC4143294; doi:10.1371/journal.pone.0105504)
Supplement: Table S4 — Nucleotide sequences of specific primers. (DOC) [file pone.0105504.s004.doc]

**Table S4** Nucleotide sequences of specific primers

| Target genes | Sequences (5’ to 3’) |  | Products | GenBank No. |
| --- | --- | --- | --- | --- |
| mRNA expression |  |  |  |  |
| *PC* | F:ccgcaagatgggagacaaggt | R:ggaagccgtaggtgttggagaa | 131 bp | NM_214349.1 |
| *PEPCK1* | F: tcagcacgactccagccttca | R: gctcaagcagtctgggcattct | 122 bp | NM_001123158 |
| *PEPCK2* | F:acaggaggttcgtgacattcgg | R: gtggtgctgtgctcacttgcta | 149 bp | XM_001928468 |
| *FBP1* | F: tccaccgcacgctggtctat | R: ccagtcctcctgccttctccat | 129 bp | NM_213979 |
| *G6PC* | F:aagccaagcgaaggtgtgagc | R: aagcattcagccaaca | 165 bp | NM_001113445 |
| *BHMT* | F:gaggctgtgtgggcagttgaag | R:acaatggatgctcctgcctttacc | 143 bp | NM_001200042.1 |
| *MAT2B* | F: tggtgttcattatttgctttgcttg | R: ggaggcatcaagctgcagga | 143 bp | NM_001142832 |
| *AHCYL1* | F: gtggtggtgtgtggctacgg | R: gcagagcacagatggggtca | 106 bp | NM_001201381.1 |
| *PPIA* | F: gactgagtggttggatgg | R: tgatcttcttgctggtctt | 116 bp | NM_214353.1 |
| *PEPCK1* promoter | F: gccgcccagcactcattaa | R: gccggtctttggatcatg | 104 bp |  |
| *PEPCK2* promoter | F: cttgagaaatcaccagagggag | R: gctcatgacaacgctgaatct | 127 bp |  |
| *FBP1* promoter | F: cctggcttaaccttctgaact | R: ccacctgcatggtttcct | 109 bp |  |
| *G6PC* promoter | F: tgaggatggaggagggaatg | R: tggaaccagatggggaaga | 210 bp |  |
| negative control | F:agtagacacccagcagattcat | R: tgaggagggacaaaggga | 103 bp |  |

AHCYL1, adenosylhomocysteine hydrolase-like 1; BHMT, betaine-homocysteine methyltransferase; FBP1, Fructose-1,6-bisphosphatase; G6PC, Glucose-6-phosphatase; MAT2B, methionine adenosyltransferase II beta; PC, Pyruvate carboxylase; PEPCK1, cytoplasmic phosphoenolpyruvate carboxykinase; PEPCK2, mitochondrional phosphoenolpyruvate carboxykinase.
