# Supplementary material for: Betaine Supplementation in Maternal Diet Modulates the Epigenetic Regulation of Hepatic Gluconeogenic Genes in Neonatal Piglets
Source: PLoS One. 2014 Aug 25;9(8):e105504. doi: 10.1371/journal.pone.0105504 (PMC4143294; doi:10.1371/journal.pone.0105504)
Supplement: Table S5 — Antibodies for this experiment. (DOC) [file pone.0105504.s005.doc]

**Table S5** Antibodies for this experiment

| Antibodies | MW | Species | Source | Catalogue no. |
| --- | --- | --- | --- | --- |
| Western blotting | | | | |
| PC | 130 kd | Rabbit | proteintech^TM^ | 16588-1-AP |
| PEPCK1 | 67 kd | Rabbit | proteintech^TM^ | 16754-1-AP |
| PEPCK2 | 71 kd | Rabbit | proteintech^TM^ | 14892-1-AP |
| FBP1 | 37 kd | Rabbit | proteintech^TM^ | 12842-1-AP |
| G6PC | 40 kd | Rabbit | Abcam | ab83690 |
| BHMT | 50 kd | Rabbit | proteintech^TM^ | 15965-1-AP |
| MAT2B | 38 kd | Rabbit | proteintech^TM^ | 15952-1-AP |
| AHCYL1 | 60 kd | Rabbit | proteintech^TM^ | 10658-3-AP |
| SETD7 | 49 kd | Rabbit | Abcam | ab114145 |
| EZH2 | 85 kd | Rabbit | proteintech^TM^ | 21800-1-AP |
| GAPDH | 36 kd | Mouse | KangChen Bio-tech | KC-5G4 |
| β-actin | 42 kd | Mouse | KangChen Bio-tech | KC-5A08 |
| ChIP |  |  |  |  |
| IgG |  | Rabbit | Millipore | 12-370 |
| H3 |  | Rabbit | Abcam | ab1791 |
| H3K4me3 |  | Rabbit | Abcam | ab8580 |
| H3K27me3 |  | Rabbit | Millipore | 17-622 |

AHCYL1, adenosylhomocysteine hydrolase-like 1; BHMT, betaine-homocysteine methyltransferase; ChIP, chromatin immunoprecipitation; EZH2, Enhancer of zeste homolog 2; FBP1, Fructose-1,6-bisphosphatase; G6PC, Glucose-6-phosphatase; H3, histone H3; H3K4me3, histone H3 lysine 4 trimethylation; H3K27me3, histone H3 lysine 27 trimethylation; MAT2B, methionine adenosyltransferase II beta; PC, Pyruvate carboxylase; PEPCK1, cytoplasmic phosphoenolpyruvate carboxykinase; PEPCK2, mitochondrional phosphoenolpyruvate carboxykinase; SETD7, SET domain-containing protein 7.
