# Supplementary material for: Betaine Supplementation in Maternal Diet Modulates the Epigenetic Regulation of Hepatic Gluconeogenic Genes in Neonatal Piglets
Source: PLoS One. 2014 Aug 25;9(8):e105504. doi: 10.1371/journal.pone.0105504 (PMC4143294; doi:10.1371/journal.pone.0105504)
Supplement: Table S6 — miRNA and the corresponding primer sequences. (DOC) [file pone.0105504.s006.doc]

**Table S6 miRNA and the corresponding primer sequences**

| Name | Sequence (5’to 3’) | miRbase Accession |
| --- | --- | --- |
| ssc-miR-103 | agcagcattgtacagggctatga | MIMAT0002154 |
| ssc-miR-107 | agcagcattgtacagggctatca | MIMAT0002155 |
| ssc-miR-16 | tagcagcacgtaaatattggcg | MIMAT0007754 |
| ssc-miR-143-3p | tgagatgaagcactgtagctc | MIMAT0013879 |
| ssc-miR-184 | tggacggagaactgataagggt | MIMAT0002127 |
| ssc-miR-185 | tggagagaaaggcagttcctga | MIMAT0007759 |
| ssc-miR-196b | taggtagtttcctgttgttggg | MIMAT0025369 |
| ssc-miR-140-3p | taccacagggtagaaccacggac | MIMAT0006786 |
| ssc-miR-424-3p | caaaacgtgaggcgctgctat | MIMAT0013921 |
| ssc-miR-129b | ctttttgcggtctgggcttgc | MIMAT0020586 |
| ssc-miR-370 | gcctgctggggtggaacctggt | MIMAT0025373 |
| ssc-miR-30b-3p | agctcggtctgaggcccctcagt | MIMAT0015269 |
| ssc-miR-423-5p | tgaggggcagagagcgagacttt | MIMAT0013880 |
| ssc-miR-92b-5p | agggacgggacgcggtgcagtgtt | MIMAT0017377 |
| oligo dT adaptor | tagagtgagtgtagcgagcacagaatt  aatacgactcactataggttttttttttttttttvn | N/A |
| universal primer | tagagtgagtgtagcgagca | N/A |
| U6 | ggcaaggatgacacgcaaat | N/A |
